# Synthetic high-density lipoproteins as targeted monotherapy for chronic lymphocytic leukemia

**DOI:** 10.18632/oncotarget.14494

**Published:** 2017-01-04

**Authors:** Kaylin M. McMahon, Cristina Scielzo, Nicholas L. Angeloni, Elad Deiss-Yehiely, Lydia Scarfo, Pamela Ranghetti, Shuo Ma, Jason Kaplan, Federica Barbaglio, Leo I. Gordon, Francis J. Giles, C. Shad Thaxton, Paolo Ghia

**Affiliations:** ^1^ Department of Urology, Feinberg School of Medicine, Northwestern University, Tarry, Chicago, IL, USA; ^2^ Università Vita-Salute San Raffaele, Milan, Italy; ^3^ Strategic Research Program On CLL and Unit of B cell Neoplasia, Division of Experimental Oncology, IRCCS San Raffaele Scientific Institute, Milan, Italy; ^4^ Robert H. Lurie Comprehensive Cancer Center of Northwestern University, Chicago, IL, USA; ^5^ Developmental Therapeutics Program of The Division of Hematology Oncology, Feinberg School of Medicine, Chicago, IL, USA; ^6^ Simpson Querrey Institute (SQI) for BioNanotechnology, Chicago, IL, USA; ^7^ International Institute for Nanotechnology, Evanston, IL, USA

**Keywords:** lipoprotein, leukemia, scavenger receptor type B-I, nanoparticle, biomaterials

## Abstract

Chronic lymphocytic leukemia (CLL) remains incurable despite the introduction of new drugs. Therapies targeting receptors and pathways active specifically in malignant B cells might provide better treatment options. For instance, in B cell lymphoma, our group has previously shown that scavenger receptor type B-1 (SR-B1), the high-affinity receptor for cholesterol-rich high-density lipoproteins (HDL), is a therapeutic target. As evidence suggests that targeting cholesterol metabolism in CLL cells may have therapeutic benefit, we examined SR-B1 expression in primary CLL cells from patients. Unlike normal B cells that do not express SR-B1, CLL cells express the receptor. As a result, we evaluated cholesterol-poor synthetic HDL nanoparticles (HDL NP), known for targeting SR-B1, as a therapy for CLL. HDL NPs potently and selectively induce apoptotic cell death in primary CLL cells. HDL NPs had no effect on normal peripheral blood mononuclear cells from healthy individuals or patients with CLL. These data implicate SR-B1 as a target in CLL and HDL NPs as targeted monotherapy for CLL.

## INTRODUCTION

Chronic lymphocytic leukemia (CLL) is the most frequent adult leukemia in western countries and continues to be incurable [1]. The clinical manifestations of CLL and corresponding patient prognoses are highly variable [2]. Multiple internal (e.g., genetic and epigenetic) and external (e.g., antigenic and microenvironmental stimuli) factors have been implicated in the pathophysiology of CLL [3]. Based on these data, more effective treatment regimens have been designed to target specific molecules responsible for the survival and expansion of the leukemic clone. However, long-term cure has not been realized and patients relapse even after these therapies indicating that further approaches are needed to enable curative therapy.

Reductions in HDL cholesterol (HDL-C) have been documented in patients with CLL. Freshly isolated CLL cells from patients were found to have reduced free cholesterol, but increased cholesteryl ester content. Treatment of the CLL cells with targeted inhibitors of cholesteryl ester formation reduced cell proliferation [4]. Furthermore, analysis of CLL cells obtained from patients revealed an increase in cellular 3-hydroxy-3methyl-glutaryl-coenzyme A reductase (HMG-CoA reductase), which is the rate-limiting enzyme for cholesterol synthesis. Additionally, data showed that CLL cells do not utilize LDL and the LDL-receptor for cholesterol uptake. Collectively, these data suggest CLL cells increase cholesterol ester content via uptake of cholesteryl ester from HDLs or by *de novo* intracellular synthesis. Cholesterol metabolism, particularly inhibition of cholesteryl ester formation and uptake, may potentially provide new therapeutic opportunities for CLL.

Many malignant cells have been shown to overexpress SR-B1, the high-affinity receptor for cholesterol-rich HDL [5–9]. Cholesterol and cholesteryl ester carried by HDLs are delivered to cancer cells through SR-B1 [10]. SR-B1 resides in plasma membrane lipid rafts [11] where it functions to maintain cholesterol balance and, in a cell-specific manner, is involved in second messenger signaling [12]. Upon binding to SR-B1, HDL facilitates bi-directional diffusion of free cholesterol between the HDL particle and the plasma membrane, and delivers cholesteryl ester from the particle core to the cell [13]. Ultimately, cholesteryl ester delivery reduces particle size and the affinity of HDL for SR-B1 whereupon the remnant particle disengages from SR-B1 [12]. Our group has explored synthetic HDL nanoparticles deplete of free cholesterol and cholesteryl ester as therapy for B cell lymphomas. The HDL NPs are synthesized using a 5 nm diameter gold nanoparticle (AuNP) to control size and shape. Because of the AuNP core, HDL NPs fail to shrink in size and bind SR-B1 more tightly relative to their cholesterol-rich natural HDL counterparts [16]. Our data demonstrate that the HDL NPs outcompete native HDL for SR-B1 and modulate cholesterol metabolism (i.e. via enhanced free cholesterol removal and reduced cholesteryl ester uptake), which potently induces apoptosis in human B cell lymphoma *in vitro* and *in vivo* without measured systemic side effects in the studied animal models [14–16].

We hypothesized that CLL cells express SR-B1 and that the HDL NP would produce a therapeutic response in primary cells isolated from patients with CLL. To test this hypothesis we first investigated SR-B1 expression in healthy peripheral blood mononuclear cells (PBMCs) and CLL cells collected from patients. We treated normal PBMCs from healthy individuals and CLL cells obtained from patients with HDL NPs and measured potential toxicity and therapeutic response, respectively. In short, our data demonstrate that, in contrast to normal B cells, CLL cells express SR-B1 and the HDL NPs potently induce apoptosis in primary CLL cells.

## RESULTS

### SR-B1 expression in PBMCs isolated from healthy volunteers

**Figure 1:**
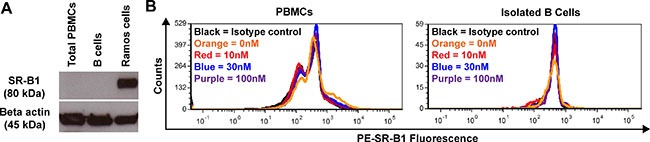
SR-B1 expression with and without HDL NP treatment (healthy volunteers) **(A)** Expression of SR-B1 and beta actin as measured by Western blot in PBMCs, B cells, and Ramos cells (positive control). **(B)** Expression of SR-B1 in PBMCs and B cells in the presence [10 nM (red), 30 nM (blue), or 100 nM (purple)] or absence [0 nM (orange)] of HDL NPs compared to isotype control (black).

We studied by Western blot the expression of SR-B1 on different leukocyte subpopulations present in the peripheral blood of healthy volunteers. Data showed that SR-B1 was not detected in lysates from total PBMC or isolated B cells (Figure 1A). Using flow cytometry, SR-B1 expression remained negative and was not modulated in total PBMCs or B cells after incubation with HDL NPs (Figure 1B). We analyzed selected subpopulations of PBMCs by flow cytometry based on side scatter (SSC) and surface marker characteristics. Weak expression of SR-B1 was detected in the presence and absence of HDL NPs in eosinophils [SSC^high^, CD45^high^, CD16^−^, CD2^+^, CRTH2^+^] and immature granulocytes [SSC^high^, CD45^+^, CD16^−^, CD2-, CRTH2^−^] (Supplementary Figure 1A, 1B). In contrast, all other subpopulations tested including CD16^+/−^ monocytes [SSC^low^, CD2^−^, CRTH2^−^, CD19^−^, CD36^+^], cytotoxic T cells [SSC^low^, CD45^high^, CD16^+^, CD2^+^, CRTH2^+^], non-cytotoxic T cells [SSC^low^, CD45^high^, CD16^−^, CD2^+^, CRTH2^+^], and myeloid progenitor cells [SSC^low^, CD45^low^, CD19^−^, CD2^−^, CRTH2^−^] did not express SR-B1 by cytofluorimetric analysis (Supplementary Figure 1C–1G). In addition, HDL NP treatment did not significantly change SR-B1 expression in subpopulations of the PBMCs analyzed (Supplementary Figure 1).

### HDL NPs are not toxic to PBMCs, B cells, or T cells isolated from healthy volunteers

**Figure 2:**
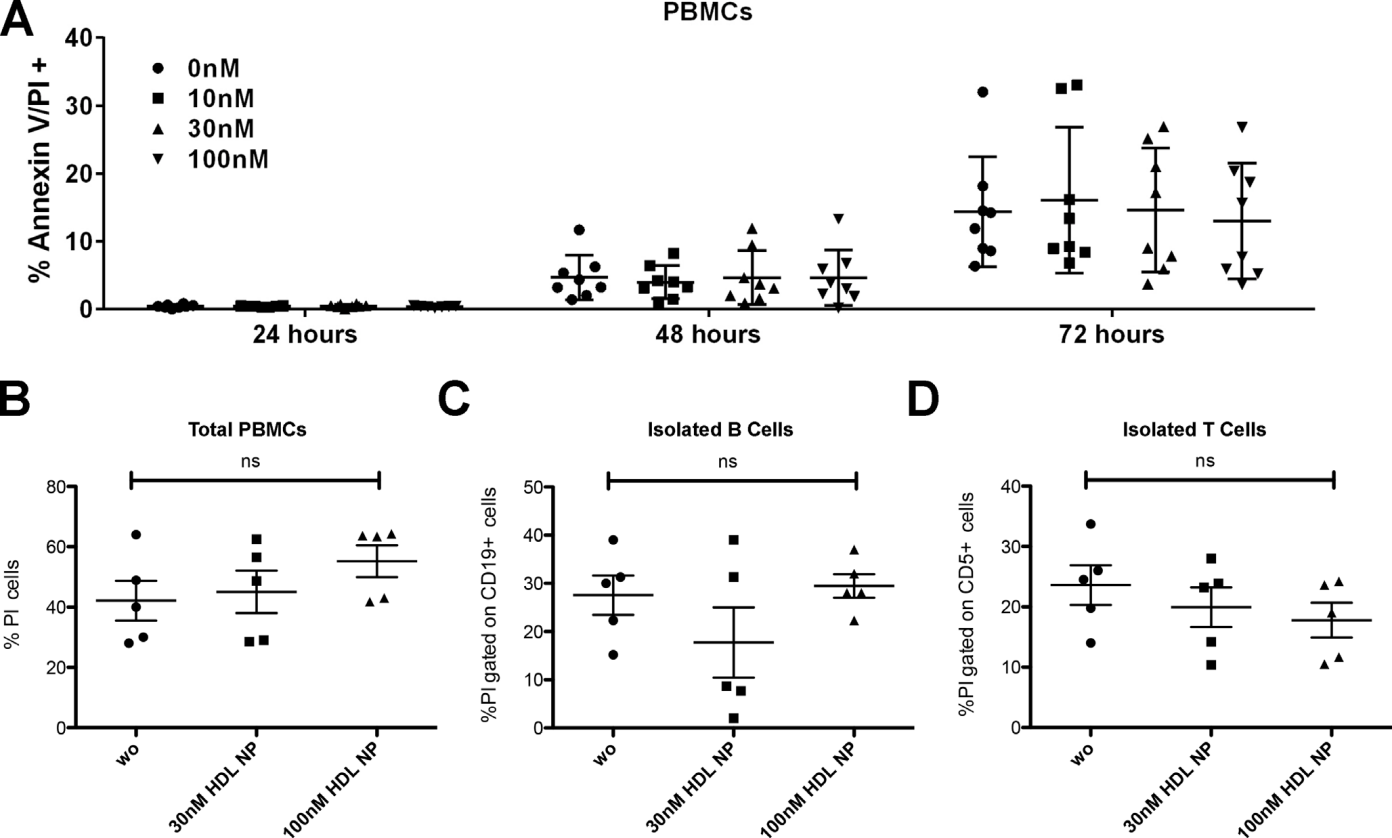
Toxicity of HDL NPs to PBMCs, B and T cells (healthy volunteers) **(A)** Cell viability of PBMCs obtained from healthy individuals in USA after treatment with 0, 10, 30, and 100 nM HDL NPs at 24, 48, and 72 hrs. Cell viability of **(B)** PBMCs, **(C)** isolated B cells, and **(D)** isolated T cells after treatment with 0 (wo), 30, or 100 nM HDL NPs for 72 hours in Italy cohort.

In order to determine the potential toxicity to healthy cells, total PBMCs were incubated with HDL NPs. In a cohort of healthy volunteers in the United States (USA), no significant difference in apoptosis was demonstrated in total PBMCs when treated with 10, 30, or 100 nM HDL NP for 24, 48, or 72 hrs (Figure 2A). Similar data were collected in samples from healthy volunteers in Italy, and showed that HDL NP were not toxic to PBMCs (Figure 2B), B cells (Figure 2C), or T cells (Figure 2D).

### Clinical characteristics of the CLL patients and SR-B1 expression in CLL patient cells

**Figure 3:**
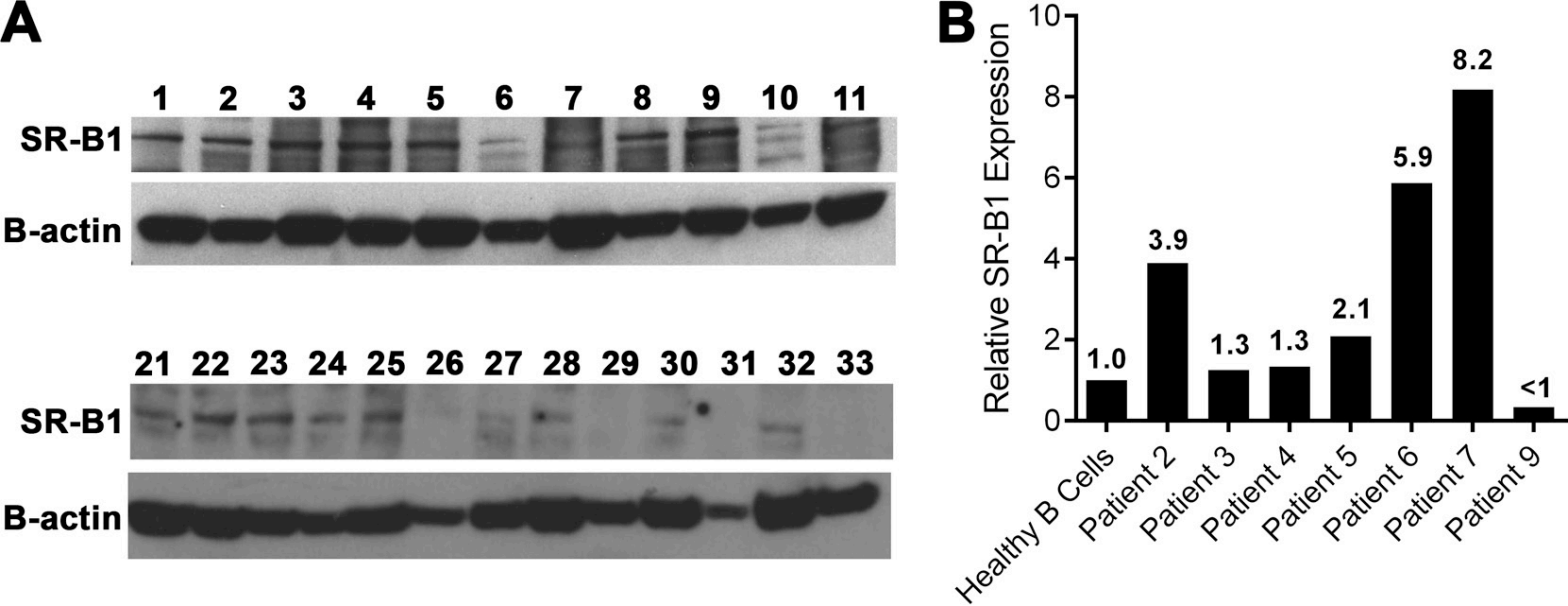
SR-B1 expression by CLL cells (patient samples) **(A)** Western blot of SR-B1 expression in primary human CLL cells from Italy. **(B)** Relative SR-B1 expression of CLL cells versus healthy B cells obtained in USA and measured by flow cytometry.

The clinical characteristics of the CLL patients can be found in Table 1. Western blot for SR-B1 was also performed on isolated CLL cells from the patient samples collected in Italy (Figure 3A). In addition, SR-B1 expression was measured using flow cytometric analysis of isolated CLL cells obtained from patients in the USA (Figure 3B).

**Table 1:** CLL patient characteristics

Patient Number	Sex	Rai	Binet	Surface CD38 Expression	ZAP-70	Cytogenetics	IGHV Mutation Status	Treatment
USA Patients								
1	F	0	A	−	−	Normal	N/A	No
2	F	2	B	+	+	17p-	N/A	No
3	F	0	A	−	−	11q-, 13q-	N/A	No
4	F	3	C	+	+	+12	Mutated	Yes
5	M	0	N/A	−	−	13q- x2, partial deletion IgH	N/A	No
6	M	0	A	−	−	13q-	Mutated	No
7	M	1	B	N/A	N/A	13q-	N/A	No
8	F	1	B	−	−	13q- x2	Mutated	No
9	F	1	B	−	+	13q-	N/A	No
Italy Patients								
1	F	0	A	−	−	13q-	Mutated	No
2	M	0	A	−	+	11q-	Unmutated	Yes
3	M	0	A	−	−	Normal	Mutated	No
4	F	0	A	−	+	Normal	Mutated	No
5	F	0	A	−	N/A	13q-	Mutated	No
6	M	0	A	−	−	13q-	Unmutated	No
7	M	0	A	−	−	13q-	Mutated	No
8	F	0	A	−	+	13q-	Mutated	Yes
9	F	0	A	−	−	13q-	Mutated	No
10	F	0	A	−	N/A	N/A	Mutated	Yes
11	F	N/A	A	−	−	N/A	Mutated	Yes
12	M	N/A	A	−	−	Normal	Mutated	No
13	F	0	A	+	N/A	Normal	Unmutated	No
14	M	0	A	−	N/A	Normal	Unmutated	No
15	F	0	A	−	N/A	Normal	Unmutated	No
16	M	0	A	−	N/A	13q-	N/A	No
17	M	1	A	+	N/A	N/A	N/A	No
18	F	0	A	+	N/A	Normal	Mutated	No
19	M	0	A	−	N/A	N/A	Unmutated	No
20	M	0	A	−	N/A	N/A	Mutated	No
21	M	N/A	B	N/A	+	13q-	Unmutated	Yes
22	M	2	A	+	−	+12	Unmutated	Yes
23	M	1	A	−	−	13q-	Mutated	No
24	F	0	A	+	+	17p-	Unmutated	Yes
25	M	0	A	+	−	17p-	Unmutated	Yes
26	M	0	A	−	−	17p-	Mutated	Yes
27	M	0	A	−	N/A	13q-	Mutated	No
28	M	1	A	−	+	+12	Mutated	No
29	F	0	A	−	N/A	17p-	Unmutated	Yes
30	M	0	A	−	−	13q-	Mutated	No
31	M	1	A	+	−	N/A	Unmutated	No
32	M	0	A	+	N/A	Normal	Unmutated	No
33	M	0	A	+	N/A	11q-	Unmutated	Yes

N/A: Not available; CD38 expression is defined + if > 20%; IGHV is considered mutated if homology to the genomic sequenced is < 98%; Intracellular ZAP-70 was measure by flow cytometry and consider + if > 30%.

### HDL NP treatment of PBMCs from CLL patients

**Figure 4:**
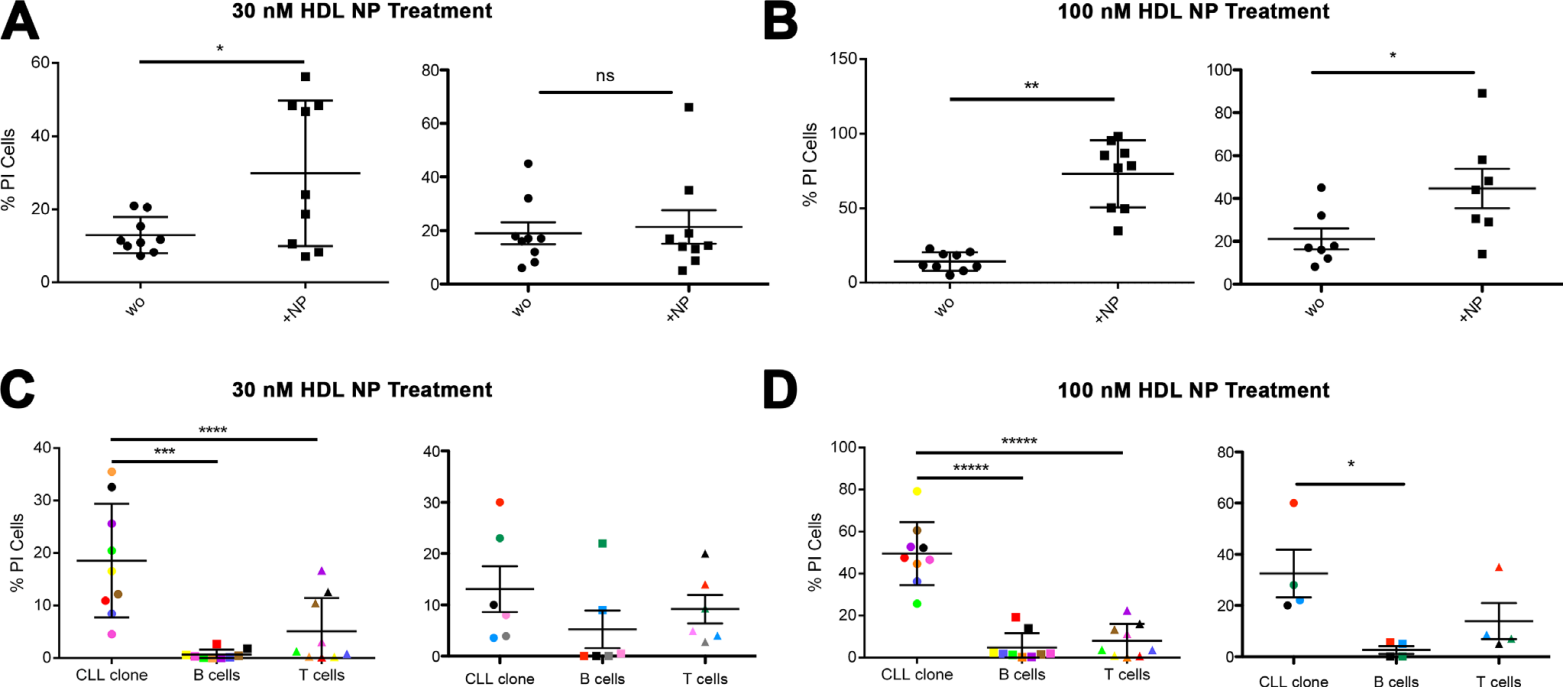
Effect of HDL NPs on CLL cells and normal B and T cells (patient samples) **(A)** Percent of apoptotic PBMCs after treatment with HDL NP (30 nM) for 72 hours (USA, left: 13.0% ± 5.0% versus 29.9% ± 19.9% *p =0.0297; Italy, right: 19.8% ± 5.0% versus 21.3% ± 6.2%). **(B)** Percent of apoptotic PBMCs after treatment with HDL NP (100 nM) for 72 hours (USA, left: 14.2% ± 6.3% versus 73.0% ± 22.5% **p = 0.0001; Italy, right: 44.7% ± 9.1%, *p = 0.043). **(C)** Percent of apoptotic CLL, B cells (gate on CD19+ CD5− cells), T cells after treatment with HDL NPs (30 nM) for 72 hours (USA, left and Italy, right). **(D)** Percent of apoptotic CLL, B cells (gate on CD19+ CD5− cells), T cells (gate on CD3+ cells) after treatment with HDL NPs (100 nM) for 72 hours (USA, left and Italy, right). For **(C)** and **(D)**, the % of CD19+CD5+ (CLL clone) cells, CD3+ cells (T cells) and CD19+CD5− B cells present in the patient's PBMC were analyzed, and the color code refers to patient number, as follows: (USA, left: Color, Patient, CLL clone %, B cell %, T cell %): Red, Patient 1, 72.93, 0.34, 2.55; Orange, Patient 2, 78.38, 3.57, 11.88; Yellow, Patient 3, 81.62, 1.14, 13.41; Green, Patient 4, 75.68, 6.51, 12.77; Blue, Patient 5, 72.55, 5.22, 6.01; Violet, Patient 6, 74.35, 1.34, 9.2; Brown, Patient 7, 72.89, 2.08, 13.07; Pink, Patient 8, 72.53, 2.37, 7.32; Black, Patient 9, 82.21, 0.75, 5.31). AND (Italy, right: Color, Patient, CLL clone %, B cell %, T cell %): Blue, Patient 3, 65, 17, 12.9; Grey, Patient 6, 85, 5, 5.2; Pink, Patient 7, 64, 8, 13.5; Green, Patient 11, 70, 14, 9; Black, Patient 15, 80, 9, 5; Red, Patient 19, 79, 3, 10.9).

Total PBMCs from CLL patients from the USA and Italy were treated *in vitro* with HDL NPs and analyzed by flow cytometry for the occurrence of apoptosis. In the USA, data demonstrate a significant increase in apoptosis when the PBMCs were treated with 30 nM HDL NP for 72 hours (Figure 4A, left). Data obtained in Italy demonstrate no significant effect after treatment with the 30 nM dose (Figure 4A, right). Treatment of PBMCs with 100 nM HDL NPs for 72 hours demonstrated significant apoptosis in both patient cohorts (Figure 4B). Importantly, viability of normal B and T cells were not affected after 30 or 100 nM HDL NP treatment in either the USA or Italian patient cohort (Figure 4C and 4D). The CLL clone from the patient cohort isolated in the USA responded to 30 nM HDL NP treatment after 72 hours (Figure 4C, left) and the CLL clones from both patient cohorts demonstrated significant apoptosis after 72 hours of 100 nM HDL NP treatment (Figure 4D).

### HDL NP treatment of malignant B cells isolated from PBMCs in CLL patients

**Figure 5:**
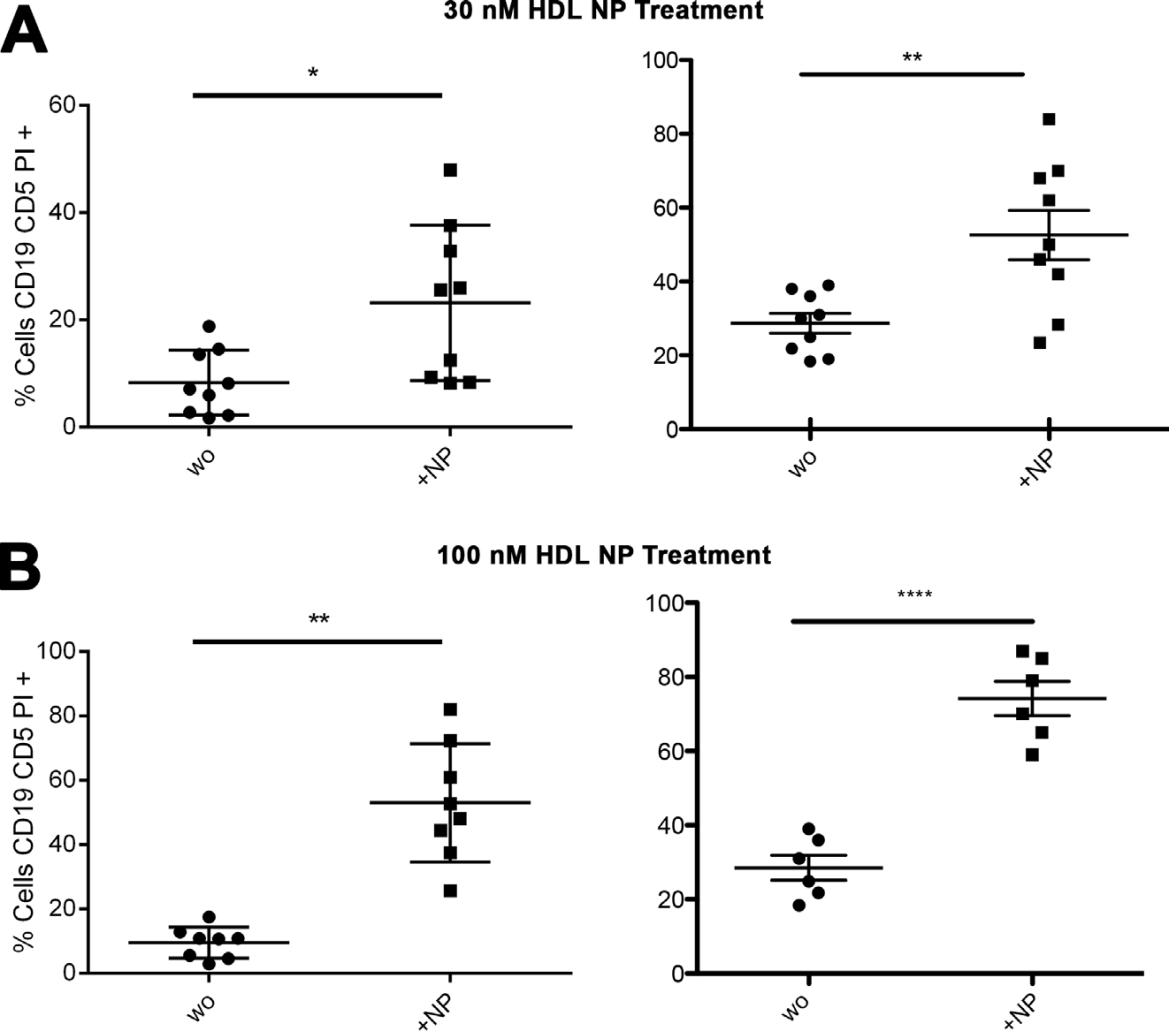
HDL NPs induce apoptosis in isolated human CLL cells **(A)** Percent of apoptotic isolated CLL cells without treatment (wo) compared to cells treated with HDL NPs (30 nM) for 72 hours (USA, left: 8.3% ± 6.1% versus 23.2% ± 14.5%; *p = 0.0059; Italy, right: 28.6% ± 2.6% versus 52.6% ± 6.7%; **p = 0.043). **(B)** Percent of apoptotic CLL cells without treatment compared to cells treated with HDL NPs (100 nM) for 72 hours (USA, left: 9.5% ± 4.8% versus 53.0% ± 18.4% **p = 0.0008; Italy, right: 28.52% ± 3.3% versus 74.2% ± 4.6%; ****p < 0.0001).

To further investigate specific killing of CLL cells, we evaluated the effect of the HDL NP (30 nM and 100 nM) on CLL cells isolated from the PBMCs. A dose-dependent pro-apoptotic effect was measured at 72 hours in samples treated at 30 nM (Figure 5A) and 100 nM HDL NP (Figure 5B) in both patient cohorts as compared to untreated controls.

## DISCUSSION

Synthetic HDL NPs are actively targeted to cancer cells by specific interaction with SR-B1 [14, 16–18]. In contrast to natural HDL, HDL NPs are highly customizable, allowing the control of many of the structural and compositional features, which confer tailored and unique functions. Functionally, compared with natural HDL, the gold template used to synthesize HDL NP enables differential manipulation of cellular cholesterol flux, promoting cellular cholesterol efflux and inhibiting the delivery of cholesterol ester [16]. The combination of SR-B1 binding and relative cholesterol starvation selectively induces lymphoma cell apoptosis *in vitro* and *in vivo* [16]. Thus, HDL NPs are targeted functional therapeutic agents enabled by the presence of a synthetic nano-template.

Data collected in human CLL suggest a requirement for cholesterol ester that is satisfied by cellular uptake of cholesterol ester-rich HDLs. Thus, we hypothesized that CLL cells would express SR-B1, and that HDL NPs would demonstrate a therapeutic response. We demonstrate that leukemic B cells from patients affected by CLL express SR-B1, and HDL NPs specifically induce apoptosis in the leukemic B cells, sparing normal lymphocytes. Indeed, our data demonstrate that SR-B1 is not expressed in normal B and T cells found in the peripheral blood, and the HDL NPs are not toxic to these lymphocytes. The HDL NPs likely induce apoptosis in CLL cells by reducing cholesteryl ester uptake through SR-B1. Further exploration of this mechanism of action is under way. These findings further expand the potential use of HDL NP as therapeutic agents for this type of leukemia.

In the USA patient cohort, HDL NPs induced apoptosis in CLL cells when the total PBMC cell population was treated with the 30 nM and 100 nM dose for 72 hours. Similar data were obtained in Italy at the 100 nM dose. In both patient cohorts, normal B and T cells were not effected by 30 or 100 nM HDL NP treatment. CLL cells isolated from PBMCs and then treated with either 30 or 100 nM HDL NPs were sensitive to treatment in both patient cohorts. Measured differences in the potency of HDL NPs to induce apoptosis in PBMCs and isolated CLL cells may be due to heterogeneity of SR-B1 expression in specific patient samples. More patient samples are required in order to better correlate SR-B1 expression with sensitivity to HDL NP treatment. Further, because a higher dose of HDL NP is more effective in PBMCs, these data suggest that there is a protective effect by the microenvironment, as shown with other drugs, [19, 20] or a dilution effect due to the uptake of HDL NP by bystander cells. In the blood of healthy individuals, flow cytometry data indicate that SR-B1 is weakly expressed by eosinophils and immature granulocytes, which may play a role in the dilution effect. However, low SR-B1 expression coupled with the low numbers of these cells in the total PBMC population results in the lack of SR-B1 signal when measured by Western blot. Further study, particularly *in vivo*, is required to elucidate the mechanism involved in HDL NP uptake in PBMCs and to determine the significance of these effects.

It is important to conceptualize whether nM doses of HDL NPs can be achieved in human patients. HDL NPs can be synthesized and concentrated into the mid μM concentration range such that reasonable volumes could be delivered to easily achieve nM dosing. In addition, intravenous administration of HDL NPs to patients with CLL represents local delivery, which increases the effective dose of the targeted HDL NP directly delivered to tumor cells. Further, studies testing the HDL NP with other therapies, including ones that also modulate cholesterol or cholesteryl ester metabolism, or tailoring the HDL NP to carry and deliver specific drugs are underway. The results may provide a potent treatment for CLL where a therapeutically active and specifically targeted “vehicle” may show enhanced properties with selectively active small molecules. In addition, our group has demonstrated the tailorability of the HDL NPs with regard to nucleic acid delivery, [21, 22] which provide a host of other opportunities for targeted delivery to CLL and other malignancies.

## MATERIALS AND METHODS

### Patients and donor samples

In Italy, fresh blood samples were obtained from patients (*n* = 33) diagnosed with CLL. CLL diagnosis was confirmed according to the National Cancer Institute Working Group (NCIWG) guidelines. [23] Patient samples were obtained after written informed consent following the protocol VIVI-CLL titled: “*In vivo* and *In vitro* Characterization on CLL” approved by the Ethical Committee at Ospedale San Raffaele, Milano, Italy. Buffy coats from anonymous healthy donors were obtained from ASST Rhodense Hospital (Rho, Italy). Patients characteristics are listed in Table 1, including biological prognostic factors such as Immunoglobulin Heavy Chain Variable genes (IGHV) mutational status and CD38 expression, evaluated as per standard procedures. All patients never received therapy or were off therapy for at least 6 months before samples were obtained. In the USA, peripheral blood samples were obtained from healthy individuals (*n* = 10, 5 male, 5 female) to perform HDL NP uptake and apoptosis assays. Informed consent was obtained and the study was approved by the Northwestern University Institutional Review Board (protocol STU00200368-MOD0001). In the USA, peripheral blood samples were obtained from individuals with CLL. Informed consent was obtained, and the study was approved by the Northwestern University Institutional Review Board (protocol NU00×3).

### B lymphocyte isolation and culture from patients and healthy volunteers

B lymphocytes expressing CD19^+^ were negatively selected from peripheral blood of patients immediately after blood withdrawal using RosetteSep B-lymphocyte enrichment kit (STEMCELL Technologies, Vancouver, BC, Canada). The purity of the cell preparations was measured using flow cytometry (FC500; Beckman Coulter, Pasadena, CA, USA) based upon cell surface co-expression of CD19 and CD5. Purity was always greater than 99%. CLL cells were CD19^+^CD5^+^, T cells were CD3+, and B cells were CD19^+^CD5^−^. Preparations were virtually devoid of natural killer (NK) cells, T lymphocytes, and monocytes. Total peripheral blood mononuclear cells (PBMCs) and purified leukemic cells were cultured in parallel in RPMI 1640 medium supplemented with 10% fetal calf serum (FCS), 2 mM L-glutamine, and 15 μg/ml gentamicin (Sigma-Aldrich, Carlsbad, CA, USA). Lymphocytes were cultured at a concentration of 1 × 10^6^ cells/500 μl in the presence or absence of different stimuli or inhibitors, as indicated. HDL NP were added to the samples at a concentration of 30 nM and 100 nM and allowed to incubate for up to 72 hours *in vitro*.

### HDL NP synthesis

Synthesis was performed as described previously. [15, 16, 22, 24].

### Western blot of SR-B1 in PBMCs, CLL cells, and B cells

Total PBMCs were isolated from whole blood from a healthy volunteer and from patients with CLL. Erythrocytes were lysed using RBC Lysis Buffer (Roche, Basel, Switzerland) according to the manufacturer's instructions. Cells were re-suspended in PBS. CLL cells were isolated per the above protocol. B cells were isolated from PBMCs using the EasySep^™^ Direct Human Naïve B Cell Isolation Kit (STEMCELL Technologies) according to the manufacturer's instructions. B cells were centrifuged (500 × *g* for 5 minutes), the supernatant was removed, protein was isolated, and western blotting performed as described previously. [18] For patient samples collected in Italy, cells were lysed with RIPA Buffer (Sigma-Aldrich, St. Louis, MO, USA) with a fresh protease and phosphatase inhibitor cocktail (Roche). Western blotting was performed using 30 μg of total protein. Membranes were incubated with 1:250 SR-B1 antibody (Abcam, Cambridge, UK) or 1:50,000 beta actin (Sigma).

### SR-B1 expression in PBMC subtypes

PBMCs were washed in PBS and re-suspended at a concentration of 1 × 10^6^ cells/ml in RPMI medium supplemented with 10% FBS and 1% penicillin/streptomycin. Cells were treated with HDL NPs (10, 30, or 100 nM) for 24 hours in vented microcentrifuge tubes in a humidified incubator, 5% CO_2_, at 37°C. The following day, cells were centrifuged (500 × *g* for 5 minutes), washed in PBS supplemented with 2% FBS, and then re-suspended in 50 μl of PBS with 2% FBS. Cells were then incubated with the antibodies listed in Supplementary Table 1 (BioLegend, San Diego, CA, USA) in accordance with a previously established protocol [25]. After a 30 min incubation at 4°C, cells were washed with PBS supplemented with 2% FBS, and then re-suspended in a total volume of 500 μl for flow cytometry analysis (Becton Dickinson (BD) LSRFortessa, Franklin Lakes, NJ, USA). Data analysis was performed using FCS Express Version 4 (DeNovo Software, Los Angeles, CA, USA). The gating strategy to identify cell types based on staining combinations was from the published literature [25]. Median fluorescent values of SR-B1 are reported, with the median values normalized to isotype control. Relative SR-B1 expression was normalize to healthy B cells.

### Toxicity assays in PBMCs from healthy volunteers

PBMCs were isolated from whole blood and re-suspended in RPMI medium as detailed above to obtain a total volume of 1.5 ml of PBMCs at 1 × 10^6^ cells/ml. Cells were treated with HDL NPs (10, 30, or 100 nM) for 24, 48, and 72 hours in vented microcentrifuge tubes in a humidified incubator, 5% CO_2_, at 37°C. Ramos cells treated with HDL NPs were subject to similar analysis as positive controls [16].

After each incubation, 500 μl of sample was retained and centrifuged (500 × *g* for 5 minutes) to recover total PBMCs. B cells were isolated from the remaining 1 ml using the EasySep^™^ Direct Human Naïve B Cell Isolation Kit according to the manufacturer's instructions. Total PBMC or B cell isolates were stained using the Annexin V-FITC Apoptosis Detection Kit (Abcam) according to the manufacturer's instructions. Flow cytometry and analysis were performed as described above.

### Viability testing of patient samples

Cell viability was assessed by Annexin V-FITC and propidium iodide (BD Biosciences) and analyzed using flow cytometry (FC500-, Beckman Coulter). CD19 ECD and CD5 PC7 antibodies (Beckman Coulter), were added to PBMCs to determine the specific effect on the CLL clone, which also allowed for analysis of CD5 expressing T cells.

### Statistical analysis

Data were compared using either the paired or unpaired Student's *t*-test. All statistical analyses were performed using GraphPad Prism version 5.0d. Statistical significance was considered significant for *P* ≤ 0.05; *denotes *P* ≤ 0.05, **P ≤ 0.01, **P ≤ 0.001, ****P ≤ 0.0001. *P*-values are two-tailed.

## SUPPLEMENTARY MATERIALS FIGURES AND TABLES


