# Supplementary material for: Synthetic high-density lipoproteins as targeted monotherapy for chronic lymphocytic leukemia
Source: Oncotarget. 2017 Jan 4;8(7):11219–27. doi: 10.18632/oncotarget.14494 (PMC5355259; doi:10.18632/oncotarget.14494)
Supplement: Supplementary file 1 [file oncotarget-08-11219-s001.pdf]

Synthetic high-density lipoproteins as targeted monotherapy for chronic lymphocytic leukemia

Supplementary Materials

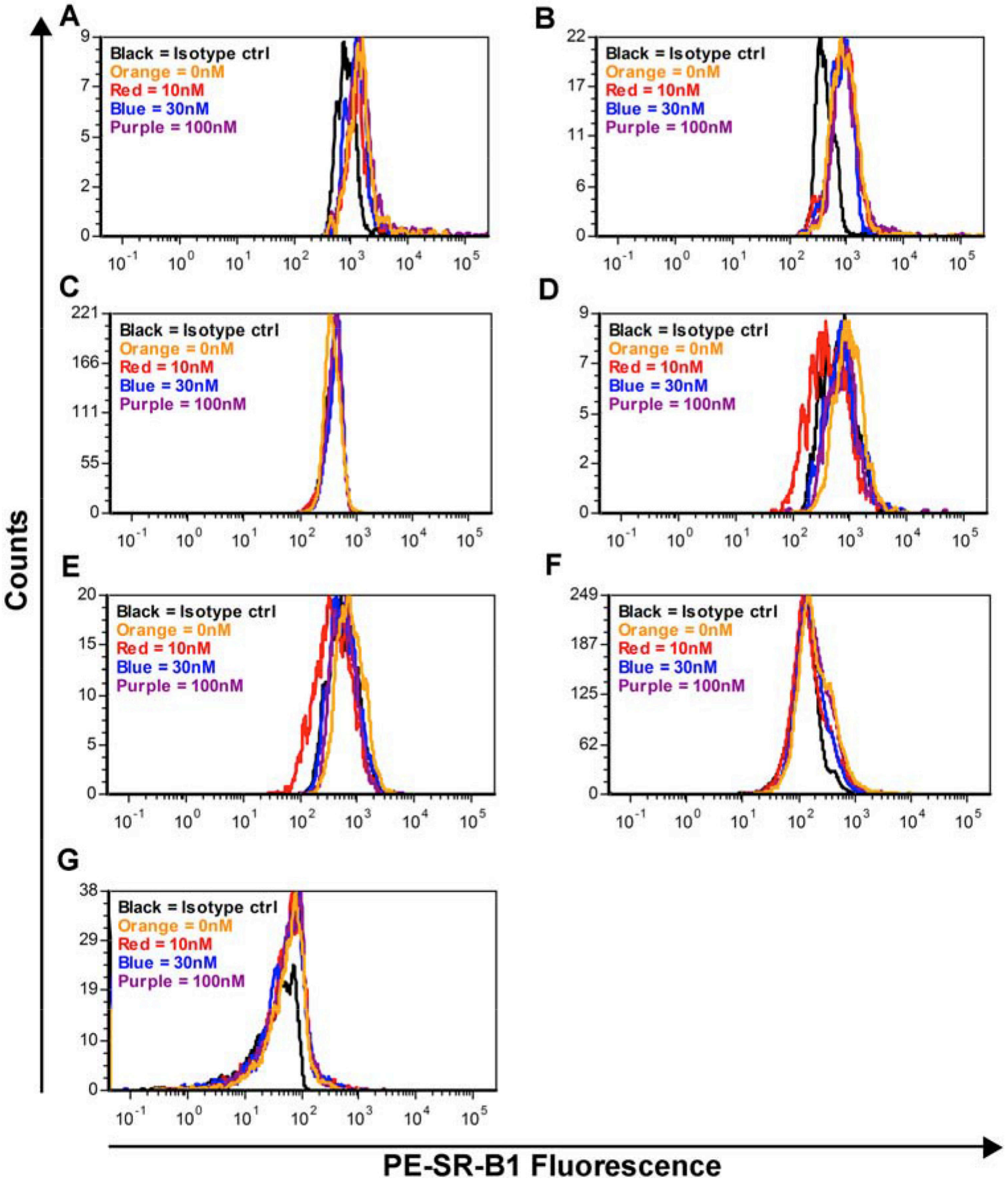

Supplementary Figure S1: SR-B1 expression in PBMC types with and without HDL NP treatment. Antibody labeling of SR-B1 expression in PBMC cell types in the presence [10 nM (red), 30 nM (blue), or 100 nM (purple)] or absence [0 nM (orange)] of HDL NPs compared to isotype control (black). (**A**) Eosinophils, (**B**) immature granulocytes, (**C**) CD16- monocytes, (**D**) CD16+ monocytes, (**E**) cytotoxic T-cells, (**F**) non-cytotoxic T-cells, and (**G**) myeloid precursor cells.

Supplemental Table 1: Fluorescent antibodies used for identification of total PBMCs by flow cytometry

Antibody	Catalog number
APC anti-human CD2	BioLegend, 300214
APC anti-human CD294 (CRTH2)	BioLegend, 350110
Brilliant Violet 711 anti-human CD16	BioLegend, 302043
FITC anti-human CD36	BioLegend, 336204
Pacific Blue anti-human CD19	BioLegend, 302224
PE/Cy7 anti-human CD45	BioLegend, 304016
PE anti-human CD36L1 (SCARB1, SR-BI)	BioLegend, 363204
